# Comparing Indications and Techniques of the Tibialis Anterior Tendon Transfer Between Clubfoot Centres in the Netherlands

**DOI:** 10.7759/cureus.106228

**Published:** 2026-03-31

**Authors:** Adam Roorda, GJFJ Bos, Christian Greve, Sophie Moerman

**Affiliations:** 1 Orthopaedic Surgery, University Medical Centre Groningen, Groningen, NLD; 2 Rehabilitation Medicine, University Medical Centre Groningen, Groningen, NLD

**Keywords:** clubfoot, guidelines, indication, postoperative care, satisfaction outcome, surgical technique, survey, tibialis anterior tendon transfer

## Abstract

Introduction

Idiopathic clubfoot is a common congenital anomaly that is treated with the Ponseti method in accordance with the guidelines showing excellent results. Nevertheless, relapse may occur for which a tibialis anterior tendon transfer (TATT) has been described as a useful treatment. However, a guideline describing the exact indication, surgical techniques and postoperative care of a TATT is not available yet. The aim of this study is to provide a survey-based overview of the pre-, peri- and postoperative steps of the various methods that are used to perform the TATT procedure throughout the Netherlands.

Materials and methods

An online survey was created, based on a literature review, and sent to the paediatric orthopaedic surgeons working at 11 different clubfoot centres in the Netherlands. We received nine complete responses from seven out of 11 (64%) orthopaedic centres. The data was collected through the online survey software Qualtrics^XM^ (Qualtrics, LLC, Provo, Utah, USA).

Results

The results showed that the TATT procedures differ from one another. How the surgical procedures were performed, in particular, varied to a great extent. However, the majority of clubfoot centres were in agreement when it comes to the age at which the TATT is performed (four to nine) and the postoperative treatment. Moreover, the respondents expressed a mean outcome satisfaction of 7.8 with very little variation.

Discussion

Based on these results, we conclude that there is a considerable variation between the TATT procedures in different clubfoot centres throughout the Netherlands. Despite this variation in execution, variation in satisfaction outcome among physicians remains limited. However, we do not know whether the satisfaction outcomes match the actual results. Thus, further research into the various methods and their results is required in order to compose adequate and standardised guidelines surrounding the TATT and optimise clubfoot care, keeping in mind that there is more than one way to bake a cake.

## Introduction

A clubfoot is one of the most common congenital deformities [1], with an incidence of 1.09 in every 1,000 live-born children in the Netherlands. In accordance with the clubfoot guideline, idiopathic clubfoot is treated in the Netherlands by the Ponseti method, during which the affected foot is manipulated step-by-step into the appropriate position by means of plaster casting and, after that, if necessary, an Achilles tendon tenotomy [2]. Although this method has excellent results, relapse after initial correction may occur in up to 48% of all cases [3]. The definition of relapse is still debated, but a commonly used definition is a recurrence of one of the four original components of a clubfoot (Cavus, Adductus, Varus or Equinus) after its initial correction [4]. It is usually treated with manipulation of the feet and application of plaster cast to maintain the correction either followed by bracing or followed by a tibialis anterior tendon transfer (TATT) [1]. During the TATT procedure, the tibialis anterior (TA) tendon is transferred to a more lateral position on the foot in order to prevent further supination during walking, thus preventing deformity and pain as well as retaining foot function [5,6]. Masrouha and Morcuende [7] described a relapse rate decrease of 53% (from 68% to 15%) after performing a TATT. When reviewing the literature, several indications for a TATT can be found, such as dynamic supination, an inversion-eversion strength imbalance and weight bearing on the lateral side of the foot [6,8-10]. Furthermore, the optimal age for a TATT has not clearly been established in the literature, while it is currently applied to children between 1.4 and 12.5 years of age [6,9-14]. Additionally, multiple surgical techniques for a TATT are used, variating in transfer technique, number of incisions [3,6-13,15-18], transfer route [3,16-18] and transfer location [6,8,9,11,12,15,16]. Finally, the literature shows that postoperatively one may opt for either lower [3,6,8,12,13,16-18] or upper [7,9,15] leg casting, both in variable durations [3,6-9,12,13,15-18].

Up until now, a consensus regarding indication, optimal technique and postoperative treatment of a TATT is missing, rendering it probable that not every physician utilises the same method in the Netherlands. This may result in suboptimal care for some clubfoot patients. Hence, the aim of this study is to provide a survey-based overview of the pre-, peri- and postoperative steps of the various methods of the TATT procedure that is applied to children suffering from clubfoot relapse within the Netherlands.

## Materials and methods

To gain insight into the TATT procedure in various clubfoot centres, an exploratory research was performed by means of a questionnaire that was sent to all paediatric orthopaedic surgeons working at one of the 11 different clubfoot centres throughout the Netherlands.

Inclusion and exclusion

The Nederlandse Orthopaedische Vereniging (NOV) has appointed 11 medical centres in the Netherlands in which all clubfoot-related care takes place. The centres are located in Amsterdam, Breda, Den Haag, Enschede, Groningen, Maastricht, Nijmegen, Rotterdam, Utrecht, Veldhoven and Zwolle. All orthopaedic surgeons employed in one of these centres who perform TATTs were approached and provided with a deadline by email via the taskforce Werkgroep Kinderorthopedie (WKO) in order to optimise the response to the survey [19,20].

Questionnaire

The literature shows no consensus regarding indication, ideal age, surgical techniques and postoperative treatment of a TATT [3,6-14,16-18]. All variations in these aspects were assessed in the questionnaire. The online survey mainly consisted of closed questions interspersed with in-depth open questions, as shown in Appendix 1. Through these questions, every step of the TATT procedure was specifically assessed. In order to ameliorate the validity of the survey and strengthen the reliability of the results, the questions were based on literature research and were tested by an expert (i.e. orthopaedic surgeon) in the University Medical Centre Groningen (UMCG) before being sent [21,22]. In order to maintain adequate reliability and comparability of the answers, every participant received the survey at the same time (May 2021). The questionnaire was created and sent to all participants using the tool Qualtrics^XM^ (Qualtrics, LLC, Provo, Utah, USA). The participants had 15 days to fill in the survey. Uncompleted questions were excluded from analysis.

Statistical analysis

Response data was collected and analysed via Qualtrics^XM^. A graphic visualising the results was composed by means of Microsoft Excel 16 (Microsoft Corporation, Redmond, Washington, USA). Answers to open questions were categorised and described as they were. Descriptive statistics, such as means, standard deviations and percentages were calculated from answers to closed questions or, if possible, from categorised answers to open questions.

Ethical statement

An Institutional Review Board (IRB) approval from the medical ethical commission (Medisch Ethische Toetsingscommissie) of the UMCG was requested and received. The approval form with reference number "M26.368181" states that the manuscript is in full agreement with the regulations of the UMCG for publication.

## Results

Out of 11 different clubfoot centres producing 22 orthopaedic surgeons performing a TATT, we received 10 (45%) responses from seven (64%) centres throughout the Netherlands. The answers of one respondent, who did not answer all questions, were excluded. The participants’ average experience as paediatric orthopaedic surgeon was 11.6 years (6.2 SD). The results are presented in various categories: indication, surgical techniques, postoperative treatment and outcome.

Indication

**Figure 1:**
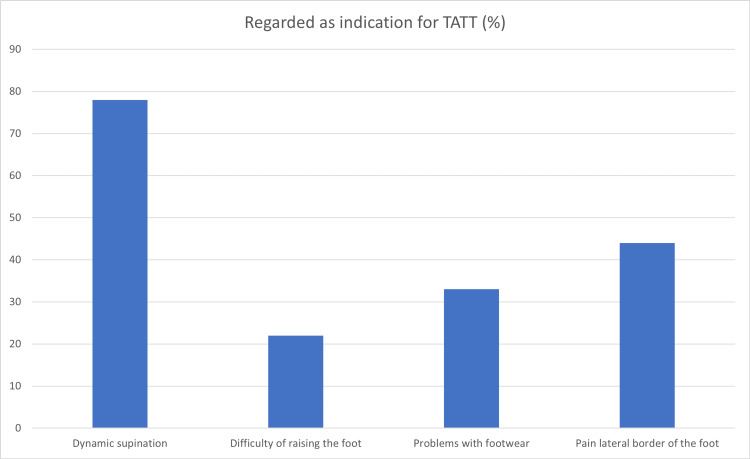
Percentage of respondents that consider various complaints or symptoms to be an indication for a TA tendon transfer TA: tibialis anterior, TATT: tibialis anterior tendon transfer.

Eight out of nine (89%) respondents usually started with a form of non-operative treatment when a clubfoot relapse was diagnosed. Multiple responses were possible, six (67%) respondents prescribed physiotherapy, five (56%) respondents applied plaster redression and the same amount used a night splint. One respondent (11%) used a brace with an abduction-dorsiflexion mechanism. Various indications for a TATT that were considered by the respondents are summarised in Figure 1. Again, multiple responses were possible.

Respondents found the optimal age for a TATT to be 5.1 years (spreading from four to eight years of age). Out of all respondents, 33% reasoned that their ideal age was based on optimal neurological and minimal anatomical maturation. Additionally, 11% provided the possibility to communicate with the patient as an argument. The remaining 56% did not elaborate their response.

Surgical techniques

Tendon Transfer Technique

Of all respondents, 44% always applied a whole tendon transfer, 22% mostly used a whole tendon transfer and 22% mostly applied a split tendon transfer. One respondent (11%) used both techniques, opting for split transposition in case of adequate peroneus muscle function. Other reported reasons for choosing a split transfer were the ability to utilise the old insertion, the absence of active pronation and the degree of dynamic forefoot adduction.

Most of the respondents (78%) consequently performed the same number of incisions during every TATT, five of which (56%) always applied the two-incision technique, while two (22%) always applied the three-incision technique. The remaining two respondents performed both techniques, depending on the rigidity of the foot and the tendon transfer technique (whole or split).

Site of Tendon Re-insertion

All respondents utilised the lateral cuneiform as site of tibialis anterior (TA) tendon insertion; four (44%) used the cuboid bone as well. One of the respondents (11%) also used the intermediate cuneiform and one reattached it to the peroneus tertius tendon (11%). Whether the respondents opted for the lateral cuneiform or cuboid depended on either perioperatively noticed anatomical accessibility (22%) or the level of deformity (11%).

Method of Tendon Fixation

Three methods of fixation were practiced by our respondents. The most popular method was attaching the tendon by means of a sponge on the plantar side of the foot which was used by 55% of the respondents. Alternative methods named were fixating the tendon by means of a bone anchor (33%) and fixating the tendon by a button over the cast (11%).

Postoperative treatment

Casting

All respondents treated their patients with a lower leg cast postoperatively. However, the duration of non-weight bearing period varied among the different practitioners with 33% allowing patients to bear weight after one week, 22% allowing weight-bearing after two weeks, 22% allowing to bear weight after four weeks and 11% allowing it after six weeks. One respondent allowed weight bearing as fast as possible, depending on the quality of fixation observed perioperatively. All respondents agreed on a total casting duration of six weeks, apart from one, who opted for a duration of eight weeks. Additional postoperative treatment was applied by 67% of all surgeons, five (56%) of which prescribed a night splint with a duration varying from six weeks to minimally six months and one (11%) did not elaborate his/her treatment.

Outcome

Follow-Up

Functional amelioration of the foot was tested by the respondents directly after surgery (22%), after six to 12 weeks (11%), after six to 12 months (22%), or at unfixed periods (22%). Observation of gait (11%), overall impression of equinus, varus and complaints (11%) and improvement of foot dorsiflexion (11%) were reported as measurements of outcome.

Satisfaction Surgeon

The participating surgeons expressed a mean outcome satisfaction of 7.8 out of 10 (0.7 SD). As far as is described, these results were based on either patient’s own satisfaction (22%), decreased supination (22%) and improved strength (22%).

Potential Improvement

Five respondents expressed their need for additional research to the following four subjects surrounding TATT: “comparing split and whole tendon transfer”, “the value of gait analysis”, “predictability of TATT functional outcomes” and “an unambiguous TATT protocol”.

## Discussion

Clubfoot care is performed by paediatric orthopaedic surgeons in 11 centres throughout the Netherlands. The aim of our study was to analyse the difference between the indication as well as execution of the TATT procedure between these centres.

Indication

Although frequently referred to as "just a number", age is of crucial importance in the procedure of a TATT. Our respondents considered the ideal age for a TATT between four and eight years, which mainly coincides with what is described in the literature [6,9-14]. Performing a TATT too early should be avoided since the child is still maturing. This may yet result in spontaneous remission. When intervening too late, on the other hand, that same skeletal maturation (soft-tissue retraction, bony changes and joint stiffness) makes the deformity more rigid with age. Another consideration to be made regarding age is the degree of ossification that has taken place at the reinsertion site in order to allow adequate interference screw fixation. Lateral cuneiform ossification centres are of sufficient size in children aged three to six years old [23]. Tendon-to-bone healing in the case of a TATT may be inadequate before that age. Furthermore, weight bearing causes the lateral foot column to grow longer than the medial column rendering the foot stiff and the deformity irreducible [24]. These possible consequences emphasize the need to analyse the optimal age for a TATT. Finally, one might consider to assess timing and amplitude of EMG activity during functional activities such as gait. For example, if the TA muscle shows no activity in late swing and hence does not generate active muscle force, a transfer to a more lateral position on the foot might be ineffective in re-storing ankle-foot in-eversion joint moment balances.

Surgical techniques

With regard to the execution of a TATT, the usage of either the "whole two-incision", "whole three-incision" or "split" technique varies greatly between the respondents, which is both remarkable and consistent with what is described in the literature [3,6-10,12,13,15-18,25,26]. Multiple studies have shown no significant difference in outcomes between these techniques, with outcomes measured as the average and maximal dorsiflexion of the ankle, level of muscle imbalance and the number of complications postoperatively [12,26]. Both our study and the literature show that either of these techniques may be used based on pre- and peri-operative findings such as the amount of forefoot pronation [10], the ability to utilise the old insertion and the absence of active pronation. Further clinical studies researching the various techniques in relation to these factors may aid surgeons in opting for the optimal transfer technique.

Furthermore, our respondents used various tendon reinsertion sites (i.e. lateral and intermediate cuneiform, cuboid and peroneus tertius tendon) based on perioperative findings or level of deformity. Hui et al. [26] describes cadaver research in which the optimal (being able to produce maximal dorsiflexion with minimal supination or pronation, all measured in amount of angular displacement) reinsertion site is shown to be the third metatarsal axis for split tendon transfer or the fourth in case of whole tendon transfer. This consideration is not shown to be made by our respondents. Moreover, translocating the TA tendon will inherently change the moment of the tendon depending among others on the reinsertion site. Further (computational modelling) research into the change of moment per insertion site may help us understand which site to choose in order to achieve optimal foot balance after a TATT.

Postoperative treatment

The literature shows that early or immediate weight bearing postoperatively does not cause complications [11-13]. Furthermore, various studies prove no increased amount of complications with early mobilisation after tendon transfer, either in the foot [27] or elsewhere [28,29]. This suggests stability of the tendon to bone interface after reinsertion and the redundancy of immobilisation after a TATT. Seeing as weight bearing does not apply axial force to the reinserted tibialis anterior tendon, the chances of tendon pull out or rupture are minimal. Nevertheless, 33% of our respondents allowed weight bearing only after four weeks probably resulting in less mobility and more muscle atrophy for their patients.

Outcome

Despite the extensive variation in indication and execution of the TATT among our respondents, a remarkable equivalence in outcome satisfaction was expressed. However, this does not imply a similar patient satisfaction. Therefore, patient satisfaction outcome after various TATT techniques should be researched, as well as objective measures of outcome such as persisting symptoms, postoperative ankle and foot function, gait assessment and amount of postoperative complications. Existing literature shows an improvement in functional outcome after a TATT. However, these correlate poorly with patient satisfaction outcome [30] which confirms how physicians and patients may differ in opinion about the final result. This inconsistency emphasizes the need for research into all outcomes. The combination of functional, patient and surgeon satisfaction outcomes may be analysed using the data that is collected by the Landelijke Registratie Orthopedische Interventies (LROI), an index created by the NOV in order to register all orthopaedic interventions [31].

Strengths and limitations

Our questionnaire was sent via an organization that is known to the respondents. A deadline was given for filling in the survey. Both measures were applied to increase the response rate. Furthermore, by means of literature-based, expertly tested questions validity and reliability of results were improved. However, our study was attenuated by a limited response from the Dutch clubfoot centres decreasing the sample, and therefore, the representativity. Just as well, the value of open questions was weakened by the absence of an inter-rater reliability. In addition, questionnaire outcomes like surgeon satisfaction are of limited value seeing as it is a subjective measurement. Moreso because it lacks validation by, for example, patient satisfaction results. Finally, one of the respondents did not answer the last four questions rendering these results inconclusive. All in all, less than half of all surgeons performing a TATT in the Netherlands responded to our survey, diminishing representativity. Therefore, the possible non-responder bias is another limitation that should be taken into consideration.

## Conclusions

Although the results of this study should be interpreted with caution as a result of the aforementioned limitations, they demonstrate that despite the overall satisfaction of surgeons with the TATT they performed, considerable variation and disagreement regarding the indication and execution of the TATT exist between orthopaedic surgeons in the Netherlands. We state that this disagreement legitimises further research into the optimal indication and execution of a TATT. If functional outcomes of various TATTs prove to be similar, one variation may be optimal based on other factors such as its costs, environmental footprint or learning curve. Taking all of these factors into account in the following research is crucial in order to create a standardised protocol providing adequate guidelines surrounding the recurrent clubfoot. The LROI database on clubfeet may provide access to large quantities of useful data by means of which our goal of adequately mapping the various methods of treating a recurrent clubfoot may be achieved.

## Appendices

Appendix 1

Questionnaire

Tibialis Anterior Tendon Transfer (TATT)

Your knowledge and experience will make a great contribution to this study, therefore we would greatly appreciate it if you would complete this questionnaire!

Completing the questionnaire will take approximately 5-10 minutes.

Thank you in advance!

**Table 1:** Questionnaire TATT: tibialis anterior tendon transfer.

No.	Question	Answer
1	At which of the Dutch clubfoot centers do you work?	
2	How many years have you been working as a paediatric orthopaedic surgeon?	
3	What percentage (0-100%) of your paediatric clubfoot patients eventually undergo a TATT?	
4	What do you consider the ideal age for a TATT and why?	
5	Is isolated dynamic supination during gait an indication for a TATT in your opinion?	○ Yes ○ No
6	Do you also consider patient complaints in the decision to perform a TATT?	○ Yes ○ No
6a	If yes: Which patient complaints are an indication for TATT?	
7	Do you usually try a conservative approach before continuing to a TATT in a recurrent clubfoot?	○ Yes ○ No
7a	If yes: Which conservative treatment(s) do you use? (Multiple answers possible)	▢ Physical therapy ▢ Plaster casting (not as TATT preparation) ▢ Night splint ▢ Other: ______
8	Do you prepare the foot with plaster casting before surgery?	○ Rarely or never ○ Sometimes ○ Often ○ Always
8a	If applicable: What is the indication for preparation by plaster casting?	
9	Do you use a full transfer or a split transfer?	○ Always full ○ Mostly full ○ Sometimes full/split ○ Mostly split ○ Always split
9a	If split: What are the criteria for choosing a split TATT?	
10	Do you use a 2-incision or 3-incision technique? (2-incision = harvest tendon and fixate laterally) (3-incision = harvest tendon, retrieve tendon from anterior compartment lower leg, fixate tendon laterally)	○ Always 2-incision ○ Always 3-incision ○ Both
10a	If both: When do you choose 2 vs. 3 incisions?	
11	How do you suture the tibialis anterior tendon?	○ Kessler ○ Bunnell ○ Whipstitch ○ Krackow ○ Other: ______
12	Do you use X-ray imaging during the procedure?	○ Yes ○ No
13	Where do you usually attach the tendon? (multiple possible)	▢ Medial cuneiform ▢ Intermediate cuneiform ▢ Lateral cuneiform ▢ Navicular ▢ Cuboid ▢ Base 3^rd^ metatarsal ▢ Base 4^th^ metatarsal ▢ Base 5^th^ metatarsal
14a	Do you always use the same attachment point?	○ Yes ○ No
14b	If no: How and when (pre- or intraoperative) do you choose the attachment point?	
15	How do you fixate the tendon?	○ Pass the tendon through to the plantar side of the foot and secure it with a sponge ○ Pass the tendon through to the plantar side of the foot and secure it with a knot ○ Using a bone anchor ○ Other: ______
16	How do you direct the tendon with respect to the extensor retinaculum?	○ Underneath the extensor retinaculum ○ Over the extensor retinaculum ○ Varies
17	How often do you combine TATT with other procedures? For instance, with an Achilles tendon lengthening or first metatarsal elevation.	○ Always ○ Often ○ Sometimes ○ Never
17a	If applicable: Is the decision preoperative or intraoperative, and based on what?	
18	What type of cast do you use postoperatively?	○ Lower leg cast ○ Above-knee cast
19	How long is the leg non-weight-bearing?	○ 1 week ○ 4 weeks ○ 6 weeks ○ Other: ______
20	How many total weeks of casting do you recommend?	○ 4 weeks ○ 6 weeks ○ Other: ______
21	Does the patient receive postoperative physical therapy?	○ Always ○ Based on indication: ______
22	Does the patient receive additional aftercare (brace/orthotics)?	○ Yes ○ No
22a	If yes: What kind and for how long?	
23	When and how do you assess functional outcome after TATT?	
24	How satisfied are you with the functional outcomes (on scale from 0–10)?	
25	Can you explain your rating?	
26	What could be improved in diagnosing recurrent clubfoot and TATT indication?	
27	Do you have additional comments regarding TATT or this questionnaire?	
